# Participant-Centered Online Active Surveillance for Adverse Events Following Vaccination in a Large Clinical Trial: Feasibility and Usability Study

**DOI:** 10.2196/14791

**Published:** 2019-10-23

**Authors:** Sally-Anne Munnoch, Patrick Cashman, Roseanne Peel, John Attia, Alexis Hure, David N Durrheim

**Affiliations:** 1 Hunter New England Local Health District Wallsend Australia; 2 University of Newcastle Newcastle Australia; 3 Hunter Medical Research Institute New Lambton Heights Australia

**Keywords:** clinical trials, active surveillance, adverse events following immunization, technology, vaccination

## Abstract

**Background:**

Active participant monitoring of adverse events following immunization (AEFI) is a recent development to improve the speed and transparency of vaccine safety postmarketing. Vaxtracker, an online tool used to monitor vaccine safety, has successfully demonstrated its usefulness in postmarketing surveillance of newly introduced childhood vaccines. However, its use in older participants, or for monitoring patients participating in large clinical trials, has not been evaluated.

**Objective:**

The objective of this study was to monitor AEFIs in older participants enrolled in the Australian Study for the Prevention through the Immunisation of Cardiovascular Events (AUSPICE) trial, and to evaluate the usefulness and effectiveness of Vaxtracker in this research setting.

**Methods:**

AUSPICE is a multicenter, randomized, placebo-controlled, double-blinded trial in which participants aged 55 to 61 years were given either the pneumococcal polysaccharide vaccine (23vPPV) or 0.9% saline placebo. Vaxtracker was used to monitor AEFIs in participants in either treatment arm through the administration of two online questionnaires. A link to each questionnaire was sent to participants via email or short message service (SMS) text message 7 and 28 days following vaccination. Data were collated and analyzed in near-real time to identify any possible safety signals indicating problems with the vaccine or placebo.

**Results:**

All 4725 AUSPICE participants were enrolled in Vaxtracker. Participant response rates for the first and final survey were 96.47% (n=4558) and 96.65% (n=4525), respectively. The online survey was completed by 90.23% (4083/4525) of Vaxtracker participants within 3 days of receiving the link. AEFIs were reported by 34.40% (805/2340) of 23vPPV recipients and 10.29% (240/2332) of placebo recipients in the 7 days following vaccination. Dominant symptoms for vaccine and placebo recipients were pain at the injection site (587/2340, 25.09%) and fatigue (103/2332, 4.42%), respectively. Females were more likely to report symptoms following vaccination with 23vPPV compared with males (433/1138, 38.05% versus 372/1202, 30.95%; *P*<.001).

**Conclusions:**

Vaxtracker is an effective tool for monitoring AEFIs in the 55 to 61 years age group. Participant response rates were high for both surveys, in both treatment arms and for each method of sending the survey. This study indicates that administration of 23vPPV was well-tolerated in this cohort. Vaxtracker has successfully demonstrated its application in the monitoring of adverse events in near-real time following vaccination in people participating in a national clinical trial.

**Trial Registration:**

Australian New Zealand Trial Registry Number (ACTRN) 12615000536561; https://www.anzctr.org.au/Trial/Registration/TrialReview.aspx?id=368506

## Introduction

Active participant-centered monitoring of adverse events following immunization (AEFI) is a recent development to improve the speed and transparency of vaccine safety postmarketing [1]. An online vaccine safety monitoring tool called Vaxtracker has demonstrated its effectiveness in active postmarketing surveillance of AEFIs in children vaccinated with the seasonal influenza vaccine [2,3]. Parents or carers of recently vaccinated children who agreed to participate were sent an email and/or SMS (short message service) text messaging with an embedded hyperlink. On responding to the link, the recipient of the message was directed to a simple, individualized online questionnaire, which could be completed via mobile phone or computer. The time between vaccination and message receipt was based on the vaccine type (live versus inactivated vaccines) and known onset time of possible symptoms associated with the vaccine. Completion of the online survey allowed for timely collation, analysis, and reporting of AEFI data, as well as detecting signals indicating possible safety concerns associated with a vaccine [2].

Vaxtracker was used to monitor AEFIs in study participants enrolled in the Australian Study for the Prevention through Immunisation of Cardiovascular Events (AUSPICE). The primary objective of AUSPICE is to determine whether the pneumococcal polysaccharide vaccine (23vPPV) is protective against cardiovascular events (fatal and nonfatal acute coronary syndrome and ischemic strokes) [4]. Participants aged 55 to 60 years at the time of recruitment with no history of cardiac or stroke events but who reported risk factors for a future cardiovascular event (ie, obesity, hypertension, or hypercholesterolemia) were recruited. Participants were randomly allocated in a double-blind trial design to either the active (23vPPV) or control (0.9% saline) treatment arms. AUSPICE participants are being followed over 6 years to determine the incidence of cardiovascular events and to compare antibody titers in response to the vaccine.

Vaxtracker has demonstrated success in safety surveillance of newly introduced childhood vaccines, including utilization and acceptance by the parents or carers of young children [2,3,5], but it has not been widely used by older populations. AUSPICE provided the opportunity to test Vaxtracker’s functionality in an older cohort of Australians, aged 55 to 60 years, participating in a large clinical trial. In Australia, 23vPPV is routinely administered to non-Indigenous adults, who are not at an increased risk of invasive pneumococcal disease, at age 65 years. Generally, 23vPPV is well-tolerated among recipients in this age group receiving the vaccine for the first time, although AEFI incidence and severity are known to increase with additional doses of the vaccine [6-8]. Symptoms commonly reported following vaccination include injection site reactions (pain, redness, swelling) and systemic events (fatigue, headache, low fever) [6,8,9]. Serious AEFIs, including cellulitis and swelling from joint to joint, have also been reported with the first and subsequent vaccine doses [7-9]. The age group for participation in AUSPICE was selected to ensure that an older cohort did not receive placebo instead of age-appropriate vaccine [4] and the risk factors required for inclusion in the study did not overlap with current recommendations for vaccine administration to prevent invasive pneumococcal disease. The vaccine is not routinely given to the 55 to 60 year age group; therefore, postlicensure safety assessments for this age group are limited, with a single study reporting injection site reactions being more common in a younger cohort (50-64 year age group) than in the usually targeted age group those aged 65 years and older [6]. Thus, monitoring the participants for adverse events following vaccination with either 23vPPV or placebo was crucial to ensure the safety of participants in AUSPICE.

There are two aims to our study: (1) to identify adverse events following vaccination with either 23vPPV or saline placebo in older persons participating in a large national clinical trial, and (2) to evaluate the usefulness and effectiveness of Vaxtracker in this research setting.

## Methods

### Study Design

The study design for AUSPICE was a multicenter, randomized, placebo-controlled, and double-blinded clinical trial. Randomization was stratified by sex and center in a 1:1 ratio for 23vPPV and 0.9% saline placebo [4]. Letters inviting people to participate in AUSPICE were sent to a random selection of residents aged 55 to 60 years who resided within a 25 km radius of one of six study sites in Australia by Medicare, Australia’s national health insurance provider [4]. To manage recruitment, enrollment, and vaccination of eligible participants, the mail-out of letters inviting people to participate was staggered over the first 22 months of the study period, commencing February 2016.

Interested participants completed an online or paper-based screening questionnaire based on study inclusion and exclusion criteria. On receipt of the completed questionnaire by the research team, participants were invited to attend their closest study clinic where their demographics, self-reported medical history, and medication information were collected, and study eligibility verified. Potential study participants were asked whether they had a previous history of pneumococcal vaccination; if so, they were excluded from the study [4].

On confirmation of eligibility, participants were allocated to one of two treatment arms via a Web-based randomization system. The active vaccine or placebo was administered intramuscularly in the deltoid region by a registered immunization nurse. Syringes for both the vaccine and placebo were similar in appearance to ensure blinding of participants [4].

### Vaxtracker

Vaxtracker was used to monitor AUSPICE participants for the occurrence of possible adverse events following vaccination with either 23vPPV or 0.9% saline solution. At the time of vaccine or placebo administration, participants were provided with information on the Vaxtracker component of AUSPICE and were encouraged to report the occurrence of immediate severe adverse events to the study site staff and to complete the Vaxtracker surveys once received. Staff emphasized the importance of completing the survey even when no symptoms were experienced following vaccination. Following double-blinded allocation and administration of the vaccine or placebo, basic demographic information, contact telephone number and email address, and vaccination details of study participants were transferred automatically from the AUSPICE database into Vaxtracker.

Seven days after vaccination, all participants enrolled in Vaxtracker were sent an email and/or SMS text message containing an embedded link that directed them to an individualized Vaxtracker survey (first survey). Seven days was used to identify known serious but rare AEFIs following vaccination with 23vPPV, including severe cellulitis and anaphylaxis, in a timely manner without compromising recall by participants [8]. This online survey requested participants to confirm their demographic details, note any chronic medical conditions, other vaccines received in the 7 days before or following the study vaccination, and to note if any symptoms were experienced in the 7 days following vaccination. If a person noted “yes” to this question, they were asked whether they had experienced any of the following 13 symptoms: redness at injection site, moderate swelling at the injections site, major swelling at the injection site (elbow joint to shoulder joint), pain at injection site, limitation of arm movement, fever, fatigue, headache, chills, rash, generalized muscle pain, generalized joint pain, or other symptoms (free text response). Noting “yes” to a symptom prompted additional symptom-specific questions, including size of swelling or redness, severity assessment of pain, extent of arm movement limitation, fever temperature, or whether the joint or muscle pain was new or aggravated. Participants were also asked whether they sought medical advice regarding the symptom selected.

Vaxtracker participants who had responded to the first survey were sent a final survey either 28 days following vaccination or immediately after submission of the first survey if the first survey was completed more than 28 days following vaccination. The final survey, again sent by either email and/or SMS text message, was designed to identify severe health events in the month following vaccination with either the active or placebo vaccine. This questionnaire asked participants whether they had been hospitalized in the previous 4 weeks and, if so, requested the admission date, diagnosis, and a preferred contact number so they could be interviewed further by study staff. All participants responding to the final survey were asked to note any other comments. We again used 28 days to identify severe but rare adverse events following vaccination with 23vPPV [6,8,10].

Participants who had not completed a survey (nonresponders) were sent two messages 3 and 6 days after the initial survey dispatch through Vaxtracker’s automated reminder program. If no response was received within 3 days of the second reminder, study site staff attempted to contact the nonrespondents by telephone.

It was recognized that a small proportion of the study population might not have access to a mobile phone or email address. Telephone interviews were conducted by study staff for Vaxtracker participants providing a landline phone number, using the same questionnaire. Interviewers were blinded to the vaccine status of the participants.

### Questionnaire Design

Symptoms used for the Vaxtracker questionnaire and time periods for message dispatch were identified through a literature review of possible adverse events following vaccination with 23vPPV [6,9-11].

**Figure 1:**
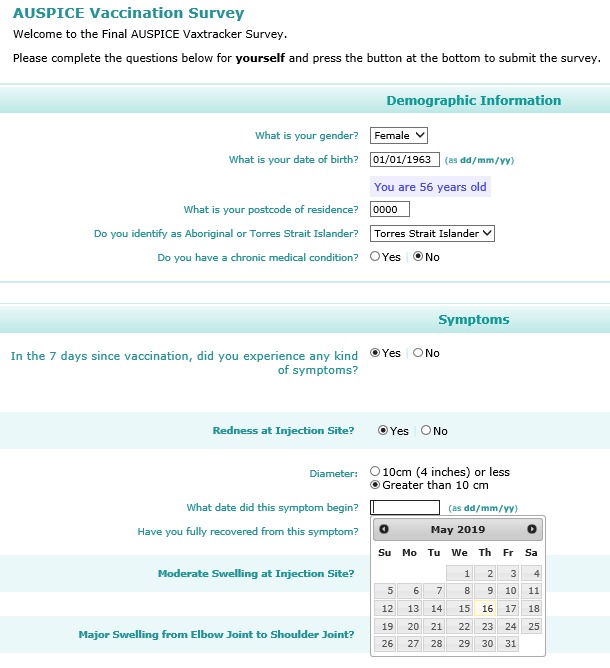
Screenshot of the main Vaxtracker survey accessed from an email via a desktop computer.

**Figure 2:**
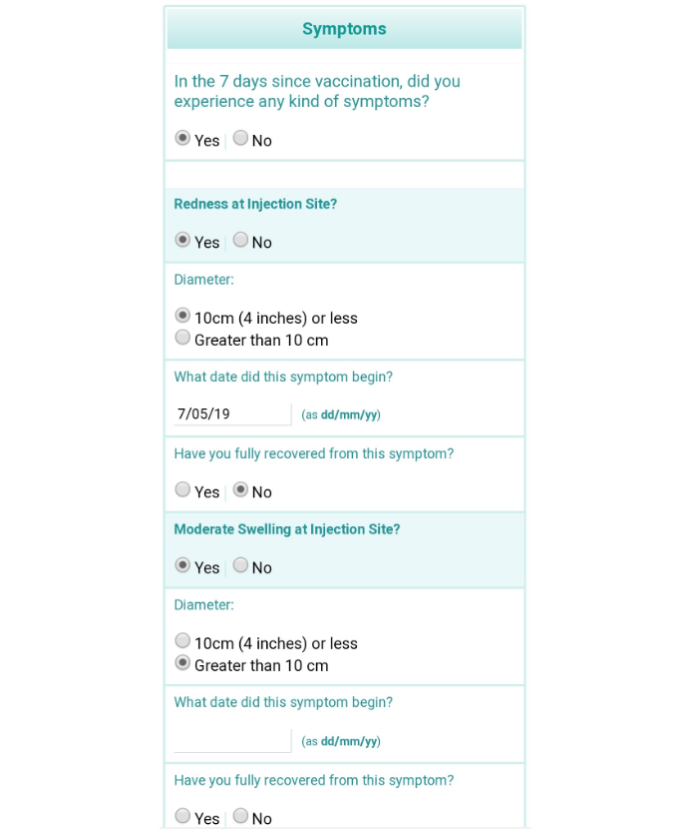
Screenshot of main Vaxtracker Survey viewed from a mobile phone.

The Vaxtracker surveys were designed to ensure easy navigation using a computer mouse, stylus, or finger, to cater for people using either a desktop or laptop computer, tablet, or mobile phone (Figures 1 and 2). A combination of drop-down fields, radio buttons, calendar control fields, and minimal free text fields were used to improve data quality.

### Severe Adverse Event Reporting and Follow-Up

AUSPICE brochures, the study’s nurse immunizers, and the Vaxtracker emails, SMS text messages, and questionnaires encouraged participants concerned about the severity of their symptoms following vaccination to seek medical advice immediately. In this instance, participants were requested to provide information about their participation in AUSPICE and possible vaccination with 23vPPV.

When a patient reported a serious symptom or medical event (extensive limb swelling, visit to an emergency department or hospital, or other serious events) via the online questionnaire, an automated serious symptom alert was forwarded to the Vaxtracker team and study staff at the center where the patient had received their vaccination. These participants were then contacted by telephone by study staff and interviewed to confirm the serious symptom, the outcome of the visit, and to provide additional counseling and information as required.

### Signal Detection

To identify possible safety signals associated with 23vPPV used for AUSPICE, we compared individual reaction rates with data reported by Jackson et al [10] comparing the safety of the 13-valent pneumococcal conjugate vaccine to the 23vPPV in pneumococcal vaccine-naïve adults. Although the age of our cohort was younger (55-60 years compared with 60-64 years) and the time period for symptom review was shorter (7 days compared with 14 days), these data were considered useful for the purposes of signal identification.

### Incorrect Contact Detail Monitoring

Email and SMS text message dispatch records were monitored weekly to identify incorrect email addresses. A dedicated Vaxtracker mailbox was used to receive “bounced” emails. Recipients of the messages were asked to respond to the Vaxtracker site if the message was received erroneously. If an error in an email address or mobile phone number was identified, survey completion logs were reviewed to check whether the survey had been completed through the alternate link, and the participant was contacted to correct the error. If the surveys had not been completed, study staff were asked to confirm contact details for participants, and if no error was identified, to contact the AUSPICE participant directly using alternative contact details (ie, home or work landline number).

### Vaxtracker Platform

The AUSPICE Vaxtracker Web-based application is built on the ASP.NET MVC framework and related Microsoft platforms [12]. It used the Application Programming Interface (API) functionality of the app, allowing for secure transfer of AUSPICE participant information to the Vaxtracker database. A unique identifier enabling the linking of AUSPICE participants to Vaxtracker was generated, ensuring that duplicates would not be inadvertently created via a system or communications error. For each enrollment request, Vaxtracker returned its own unique identifier for the new participant back to AUSPICE, permitting full two-way cross-linkage between the datasets of the two systems.

### Analyses

Descriptive analyses and statistical calculations were conducted in Excel 2010 (Microsoft Corporation, Redmond, WA, USA) and Stata 15 (StataCorp LP, College Station, TX, USA). Differences in proportions were compared using chi-square tests. A .05 level of significance was used for all analyses.

### Ethics and Funding

The University of Newcastle (H-2014-0064) and Hunter New England Human Research Ethics Committees (15/08/19/3.01) were the lead ethics committees for the AUSPICE trial, including approval for the monitoring of adverse events using Vaxtracker. Further ethics approvals or registration occurred with each of the local HRECs for all AUSPICE study centers. AUSPICE was funded by the National Health and Medical Research Council of Australia.

## Results

We enrolled all 4725 AUSPICE participants in the Vaxtracker component of the study. The period for study enrollment was from February 2016 to December 2017. The median age of participants in Vaxtracker was 58.1 years (range 55-61 years) with males representing 51.47% (2432/4725) of the cohort. A small number (n=38) of participants older than 60 years were recruited into the study because their birthday fell within the period between study registration and vaccination. There were slightly more participants randomized to the treatment arm of the trial than the placebo arm (2368/4725, 50.12% versus 2357/4725, 49.88%)

The overall response rates (both participant-completed and study site-completed surveys) to the first and final Vaxtracker surveys were 99.10% (4682/4725) and 99.27% (4648/4648), respectively. The participant completion rate for both surveys was 96.47% (4558/4725) and 96.65% (4525/4682). There were no statistically significant differences in participant response rate by treatment arm for the first survey (vaccine: 2290/2368, 97.71%; placebo: 2268/2357, 96.22%; *P*=.37) or final survey (vaccine: 2268/2346, 96.68%; placebo: 2257/2336, 96.62%; *P*=.91). When reviewing response rate by gender, females were more likely to respond to the first survey compared with males (first survey: 2228/2293, 97.17% versus 2330/2432, 95.81%; *P=.*01). Females were also more likely to complete the final survey when compared with males; however, this difference was not statistically significant (Table 1).

The times from link dispatch (via email or SMS text message) to survey submission were calculated for participant-completed questionnaires. The median time from dispatch to main survey submission was 4 hours (range 0.05 hours-47 days), with 90% (4103/4558) of surveys completed within 71 hours of the link being sent. For the final survey, the median time between dispatch and submission was 5 hours (range 0.03 hours-74 days) with 90% (4072/4525) of surveys completed within 72 hours of dispatch.

Although the majority of participants provided both an email address and mobile number, when comparing the method by which the link was sent (via email or via SMS text message), AUSPICE Vaxtracker participants were more likely to respond via a link sent by email for both surveys and when stratifying by gender (*P*<.001). Males were more likely to respond via link sent by email for the final survey compared with females (1525/2323, 66.65% versus 1368/2207, 61.98%; *P=.*01). Males were also more likely to respond from a desktop or laptop computer for both surveys compared with females, whereas females preferred to respond via their mobile device for both surveys (Table 1).

**Table 1:** Response rates to the AUSPICE Vaxtracker first (N=4725) and final survey (N=4682).

Survey attributes by survey type			Male, n (%)	Female, n (%)	*P* value
**First survey**					
	Number of people enrolled		2432 (51.47)	2293 (48.53)	—^a^
	Overall response rate		2403 (98.81)	2279 (99.39)	.04
	Response rate by participants		2330 (95.81)	2228 (97.17)	.01
	**Response rate via link type**				
		Email link	1440 (61.38)	1333 (60.15)	.40
		SMS^b^ text message link	890 (38.54)	895 (40.77)	.13
	**By device type**				
		Mobile device (mobile phone/tablet)	1151 (47.29)	1337 (58.31)	<.001
		Computer (desktop/laptop)	1179 (48.48)	891 (38.86)	<.001
**Final survey^c^**					
	Number of people eligible for final survey		2403 (51.32)	2279 (48.68)	—
	Overall response rate		2387 (99.33)	2261 (99.21)	.63
	Response rate by participants		2313 (96.25)	2212 (97.06)	.12
	**Response via link type**				
		Email link	1525 (66.65)	1368 (61.98)	.01
		SMS text message link	788 (34.44)	844 (38.66)	.003
	**By device type**				
		Mobile device (mobile phone/tablet)	1104 (45.94)	1369 (60.07)	<.001
		Computer (desktop/laptop)	1209 (50.31)	843 (33.99)	<.001

^a^Not applicable.

^b^SMS: short message service.

^c^Only participants completing the first survey, online or via a study site staff member, received the second survey.

Adverse events following immunization were reported by 34.40% (805/2340) of 23vPPV recipients in the 7 days following vaccination (Table 2). Symptoms most often reported by vaccine recipients were pain at injection site (587/2340, 25.09%), limited arm movement (224/2340, 9.57%), and generalized muscle pain (217/2340, 9.27%). In total, 240 of 2332 (10.29%) placebo recipients reported symptoms following vaccination; fatigue and headaches were the dominant symptoms reported (103/2332, 4.42%; 98/2234, 4.20%, respectively). When reviewing participants reporting symptoms by vaccine, a higher proportion of placebo recipients sought medical advice in the 7 days following vaccination compared with 23vPPV recipients (18/240, 7.5% versus 15/805, 1.9%, *P=.*001). One patient reported visiting an emergency department in the 7 days following vaccination with the placebo for an unrelated reason.

**Table 2:** Symptom profiles for participants receiving 23vPPV and placebo in the seven days following vaccination

Symptoms	23vPPV (N=2340), n (%)	Saline placebo (N=2332), n (%)	*P* value
Any symptom	805 (34.40)	240 (10.30)	<.001
Any local symptoms	660 (28.21)	59 (2.53)	<.001
Redness at injection site	146 (6.24)	12 (0.51)	<.001
Swelling at injection site	207 (8.85)	12 (0.51)	<.001
Pain at injection site	587 (25.09)	49 (2.10)	<.001
Limited arm movement	224 (9.57)	11 (0.47)	<.001
Any systemic symptom	438 (18.72)	180 (7.72)	<.001
Fever (reported)	81 (3.46)	54 (2.32)	.02
Fatigue	199 (8.50)	103 (4.42)	<.001
Headache	172 (7.35)	98 (4.20)	<.001
Chills	56 (2.39)	30 (1.29)	<.001
Rash	31 (1.32)	3 (0.13)	<.001
Extensive arm swelling^a^	13 (0.55)	0 (0.0)	<.001
Generalized muscle pain	217 (9.27)	64 (2.74)	<.001
Generalized joint pain	50 (2.14)	32 (1.37)	.047
Medical advice sought (symptomatic patients)^b^	15 (1.9)	18 (7.5)	<.001

^a^Described as elbow joint to shoulder joint.

^b^23vPPV: N=790, saline placebo: N=240.

When comparing AEFI rates, females were more likely to report any type of reaction for both the vaccine and placebo when compared to males (vaccine: 433/1138, 38.05% versus 372/1202, 30.95%; *P*<.001; placebo: 141/1139, 12.38% versus 99/1193, 8.30%; *P*=.001) in the 7 days following vaccination. When stratifying reaction rates into broad reaction types for vaccine recipients (any local symptoms, any systemic symptoms), the difference in symptom rates between gender still applied (Table 3). Similarly, females receiving the placebo were more likely to report any symptoms (141/1139, 12.38% versus 99/1193, 8.30%; *P*=.001) and any systemic symptoms (105/1139, 9.22% versus 75/1193, 6.29%; *P=.*008) compared with males; however, this difference was not statistically significant for the reporting of any local symptoms. When reviewing individual symptom rates, females were more likely to report most individual symptoms following vaccination compared with males (Table 3) for both the placebo and the vaccine. Females were also more likely to report extensive arm swelling from elbow to shoulder joint (10/1138, 0.88% versus 3/.25, 0.25%; *P*=.04) after vaccination with 23vPPV compared with males (Table 3), although none of the patients reporting this symptom sought medical advice in the 7 days following vaccination. Males were more likely to report the symptom of fever (not measured) for both the vaccine and the placebo; however, this difference was not significant.

Vaccine recipients were more likely to report an AEFI during an interview by a study site member compared with those who completed a survey online (26/123, 47.3% versus 779/4559, 34.09%; *P=.*04). The overall AEFI rates for placebo recipients were slightly higher for those who were interviewed by study site staff; however, this difference was not statistically significant. When stratifying AEFI rates by gender and method of completion (interviewed by study site versus completed survey online), male vaccine recipients who were interviewed by study site staff were more likely to report an AEFI compared with females and compared with both male and female placebo recipients (Table 4).

**Table 3:** Symptom profiles for participants receiving 23vPPV and placebo in the seven days following vaccination by gender.

Symptoms	23vPPV (n=2340)			Saline placebo (n=2332)		
	Female (n=1138), n (%)	Male (n=1202), n (%)	*P* value	Female (n=1139), n (%)	Male (n=1193), n (%)	*P* value
Any symptom	433 (38.05)	372 (30.95)	<.001	141(12.38)	99 (8.30)	.001
Any local symptoms	363 (31.90)	297 (24.71)	<.001	36 (3.16)	23 (1.93)	.06
Redness at injection site	89 (7.82)	57 (4.74)	.002	10 (0.88)	2 (0.17)	.02
Swelling at injection site	120 (10.54)	87 (7.24)	.005	10 (0.88)	2 (0.17)	.02
Pain at injection site	321 (28.21)	266 (22.13)	.001	28 (2.46)	21 (1.76)	.24
Limited arm movement	157 (13.80)	67 (5.57)	<.001	8 (0.70)	3 (0.25)	.11
Any systemic symptom	236 (20.74)	201 (16.72)	.01	105 (9.22)	75 (6.29)	.008
Fever (reported)	31 (2.72)	50 (4.16)	.06	23 (2.02)	31 (2.60)	.35
Fatigue	109 (9.58)	90 (7.49)	.07	64 (5.62)	39 (3.27)	.01
Headache	97 (8.52)	75 (6.24)	.03	63 (5.53)	35 (2.93)	.002
Chills	30 (2.64)	26 (2.16)	.45	18 (1.58)	12 (1.01)	.22
Rash	23 (2.02)	8 (0.67)	.004	1 (0.09)	2 (0.17)	.59
Extensive arm swelling^a^	10 (0.88)	3 (0.25)	.04	0 (0.00)	0 (0.00)	
Generalized muscle pain	114 (10.02)	103 (8.57)	.23	48 (4.21)	16 (1.34)	<.001
Generalized joint pain	29 (2.55)	21 (1.75)	.18	21 (1.84)	11 (0.92)	.056

^a^Described as elbow joint to shoulder joint.

**Table 4:** Reported symptoms following immunization by method of survey completion (participant or study site staff member) and gender.

Method, vaccine type, and gender			Participant-completed surveys (n=4549)^a^, n (%)	Study site-completed surveys (n=123)^b^, n (%)	*P* value
**Overall AEFI^c^ rate**					
	23vPPV recipients		779 (34.09)	26 (47.27)	.04
	Saline recipients		232 (10.25)	8 (11.76)	.69
**AEFI rate by gender**					
	**Female**				
		23vPPV recipients	424 (37.89)	9 (47.37)	.40
		Saline recipients	136 (12.3)	5 (15.6)	.57
	**Male**				
		23vPPV recipients	355 (30.45)	17 (47.22)	.03
		Saline recipients	96 (8.3)	3 (8.3)	.99

^a^Denominators: F=2323, M=2226. Vaccine recipients by gender. 23V PPV: M=1166, F=1119. Saline: F=1107, M=1157.

^b^Denominators: F=51, M=72. Vaccine doses by gender. 23V PPV: M=36, F=19. Saline: F=32, M=36.

^c^AEFI: adverse events following immunization.

## Discussion

Vaxtracker proved useful for monitoring adverse events in an older cohort participating in a randomized controlled trial of vaccines, with high participation rates for all study sites, age groups, and genders using both mobile devices and/or stand-alone computers. Over 90% of AUSPICE Vaxtracker participants completed both surveys within three days of receipt of the survey link, with a median time of 4 hours (0.05 hours-54 days) and 5 hours (0.03 hours-74 days) from receipt of survey link for the main and final surveys, respectively, demonstrating Vaxtracker’s potential for rapidly detecting adverse events in close to “real time.”

Overall, 23vPPV was well-tolerated in study participants enrolled in AUSPICE, with individual reaction rates lower than those reported by Jackson et al [10], noting the differences in reporting timeframes (7 days compared with 14 days postvaccination) and the age of the cohort. Interestingly, our study identified a statistically significant difference in overall AEFI rates in patients who were interviewed by study site staff members when compared with participants completing their own surveys online. Stratifying by gender, this statistically significant difference was identified only in men who were interviewed by study site staff. Median survey completion times (from vaccination date to survey completion) was 7 days for responding participants and 19 days for participants interviewed by study site (noting that nonresponders were proactively followed up by study sites after two reminder messages, or 16 days after vaccination). Although this difference warrants consideration when comparing methods of survey completion and AEFI rates, the small number of participants interviewed (N=123) and apparent recall bias as a result of delays in survey submission should not be discounted. Despite these differences, Vaxtracker provides reassuring safety data should the AUSPICE study confirm the protective benefits of earlier pneumococcal vaccination to reduce cardiovascular events.

A finding from our study was that females were consistently more likely than males to report an AEFI following vaccination with 23vPPV based on individual symptoms (redness, swelling, or pain at the injection site; extensive arm swelling from joint to joint; limited arm movement; headache and rash) and broad symptom type (any reaction, any local reaction, and any systemic reaction). Higher AEFI rates in females have been reported previously, with heightened reactogenicity to vaccination likely due to biological (heightened immune response), social, and behavioral factors [13-15]. Sex differentials for patient outcome reporting and active vaccine safety surveillance require further investigation.

When reviewing the preferred method of response to the Vaxtracker surveys, our study identified that females in the age group of 55 to 61 years were slightly more likely to respond to an online questionnaire via a mobile device (mobile phone, tablet) compared with males. The preference of responding to a questionnaire link by computer may relate to the nature of employment or employment status; however, this cannot be confirmed because occupation and employment status were not collected in this study. The gender preference for a device type (computer versus mobile device) is an important finding from this study because it indicates that, for optimal response rates, online surveys should be sent to both email and SMS text message services, with questionnaires designed for both device types.

As expected, there were statistically significant differences in individual symptom reaction rates between the vaccine and placebo groups. By monitoring “adverse events following vaccination” in the blinded saline placebo arm, background symptom levels could be assessed and a true difference with the vaccine arm occurring in the community for this age group during the surveillance period could be determined. Background levels of symptoms occurring in the community need to be considered when interpreting postlicensure AEFI surveillance data.

A limitation of this study is that a formal cost evaluation of AUSPICE Vaxtracker has not yet been conducted. Descriptive analysis of the number of messages automatically dispatched to Vaxtracker participants was conducted, with approximately 17,200 emails and 16,900 SMS text messages to the 4725 people enrolled in Vaxtracker. In addition, 1350 emails noting serious symptom alerts were sent via email to AUSPICE and Vaxtracker staff. Study staff interviewed 247 participants when questionnaires had not been completed (due to no access to an email address or mobile phone, or those who had not responded to the questionnaire), and 82 participants when a serious symptom alert had been noted. These data suggest that Vaxtracker was an efficient method for monitoring AEFI in people participating in a large clinical trial. When considering the convenience of completing a survey by participants at a time suited to them, the usefulness of Vaxtracker in collecting timely AEFI data is obvious.

Postlicensure AEFI surveillance in near-real time is critical for monitoring the introduction of new vaccines into the community and providing assurance to people participating in research in which the additional benefits of a vaccine are being explored. For a participant-centered surveillance system to be successful in the detection of vaccine safety signals, the program needs to consider the target audience. Flexibility that caters for both mobile devices and stand-alone computer systems increases the system’s usability across age groups, as shown in this study. High response rates by older people participating in AUSPICE indicates a high level of acceptance of Vaxtracker and demonstrates the program’s effectiveness in monitoring vaccine safety in people aged 55 to 60 years. Finally, the use of automated email and SMS text message to send links to surveys provides a timely and convenient method of collecting data from people participating in large clinical trials.

## Abbreviations

AEFI: adverse events following immunization
AUSPICE: Australian Study for the Prevention through Immunisation of Cardiovascular Events
SMS: short message service
